# A superior tool for predicting sepsis in SAH patients: The nomogram outperforms SOFA score

**DOI:** 10.1371/journal.pone.0316029

**Published:** 2025-01-23

**Authors:** Lei Yu, Shan Zou, Qingshan Zhou, Beibei Cheng, Jun Jin

**Affiliations:** 1 Jinan University, Guangzhou, China; 2 Department of Intensive Care Unit, The University of Hong Kong-Shenzhen Hospital, Shenzhen, China; Chinese Academy of Medical Sciences and Peking Union Medical College, CHINA

## Abstract

**Objective:**

This study aimed to develop and validate a nomogram to predict the risk of sepsis in non-traumatic subarachnoid hemorrhage (SAH) patients using data from the MIMIC-IV database.

**Methods:**

A total of 803 SAH patients meeting the inclusion criteria were randomly divided into a training set (563 cases) and a validation set (240 cases). Independent prognostic factors were identified through forward stepwise logistic regression, and a nomogram was created based on these factors. The discriminative ability of the nomogram was assessed using the area under the receiver operating characteristic curve (AUC) and compared with the SOFA score. The model’s consistency was evaluated using the C-index, and the improvement in performance over the SOFA score was calculated using integrated discrimination improvement (IDI) and net reclassification improvement (NRI).

**Results:**

Five independent predictive factors were identified through LASSO regression analysis: mechanical ventilation, hyperlipidemia, temperature, white blood cell count, and red blood cell count. The AUC of the nomogram in the training and validation sets were 0.854 and 0.824, respectively, both higher than the SOFA score. NRI and IDI results indicated that the nomogram outperformed the SOFA score in identifying sepsis risk. Calibration curves and the Hosmer-Lemeshow test demonstrated good calibration of the nomogram. Decision curve analysis showed that the nomogram had higher net benefit in clinical application.

**Conclusion:**

The nomogram developed in this study performed excellently in predicting the risk of sepsis in SAH patients, surpassing the traditional SOFA scoring system, and has significant clinical application value.

## 1. Background

Sepsis, a life-threatening organ dysfunction resulting from dysregulated host responses to infection, poses a significant global health challenge, impacting millions of individuals worldwide annually with alarmingly high mortality rates. Timely implementation of targeted interventions can enhance the prognosis [1, 2]. The initial clinical signs of sepsis are nonspecific, and the disease progresses rapidly [3]. Despite the absence of an effective treatment, which contributes to the elevated mortality rate among sepsis patients [4], a thorough comprehension of the pathogenesis of sepsis is imperative for successful prevention and treatment. Currently, there is no established theoretical framework explaining the pathogenesis of sepsis [5]. Therefore, in clinical practice, it is essential to conduct comprehensive research on risk factors to manage infections and prevent sepsis [6].

Subarachnoid hemorrhage (SAH) ranks as the third most common type of stroke and represents an extremely severe condition. The Global Burden of Disease study revealed a substantial increase in annual stroke incidence and mortality from 1990 to 2019 [7]. In China, the stroke burden is particularly severe, with an estimated incidence of 246.8 per 100,000 and mortality of 149.5 per 100,000 in 2020, The China Stroke Surveillance Report 2021 indicates that stroke remains a leading cause of death, accounting for 22.3% of all deaths in China [8]. These statistics underscore the critical need for stroke-related research, especially for severe subtypes like SAH. Approximately one-fourth of SAH patients succumb before hospital admission; while the prognosis improves for hospitalized patients, their quality of life remains significantly diminished for many years, substantially impacting public health [9]. Various factors influence the prognosis of SAH, extending beyond the direct impact on brain tissue and associated complications like rebleeding, delayed cerebral ischemia, and hydrocephalus [10]. Evidence indicates that additional complications, such as sepsis, play a crucial role in the post-SAH prognosis. The mortality rate attributed to complications like sepsis is comparable to that resulting from the direct effects of the initial hemorrhage, rebleeding, and vasospasm [11].

Patients with subarachnoid hemorrhage (SAH) often experience fever, which can worsen cerebral hypoxia and is linked to unfavorable outcomes. Fever can manifest with or without an infection, potentially leading to unnecessary antibiotic administration in some cases [9]. Nomograms, which are graphical tools based on statistical models, are used to predict the likelihood of a specific clinical event occurring in an individual [12]. However, there is limited information on nomograms for forecasting sepsis risk in SAH patients. This study aimed to create a nomogram that can forecast the likelihood of sepsis in SAH patients, with the goal of improving clinical decision-making.

## 2. Materials and methods

### 2.1 Data source

The study utilized the MIMIC-IV (Medical Information Mart for Intensive Care-IV) database, specifically version 2.2 (2023.1.6), which contains real data from the ICU of Beth Israel Deaconess Medical Center spanning from 2008 to 2019 [13]. Permission to access and use the database was obtained with certificate number 55510406. Ethical review and approval was not required for the study on human participants in accordance with the local legislation and institutional requirements. Written informed consent from the patients/participants or patients/participants’ legal guardian/next of kin was not required to participate in this study in accordance with the national legislation and the institutional requirements.

### 2.2. Study population

The diagnostic criteria for sepsis in this study aligned with the Sepsis 3.0 criteria. Inclusion criteria consisted of individuals aged 18 years or older, diagnosed with aneurysmal subarachnoid hemorrhage, with an ICU stay exceeding 24 hours, first ICU admission, and first instance of patient information. Exclusion criteria included cases of subarachnoid hemorrhage from other causes and pregnant or lactating women.

### 2.3. Study methods

Using Navicat, the researchers extracted 34 variables at ICU admission via SQL, encompassing baseline data such as age, gender, race, BMI, and comorbidities; vital signs like systolic blood pressure, diastolic blood pressure, mean arterial pressure, heart rate, respiratory rate, pulse oximetry (SpO2), and temperature; laboratory tests including white blood cells, red blood cells, platelets, hemoglobin, red cell distribution width, electrolytes, blood glucose, anion gap, prothrombin time, partial thromboplastin time, international normalized ratio, creatinine, blood urea nitrogen, blood gas analysis; scoring indicators like SOFA score; and whether mechanical ventilation was utilized. The primary outcome of interest was the development of sepsis. Continuous variables utilized the initial measurement value at ICU admission, while categorical variables underwent preprocessing before model entry.

### 2.4. Statistical methods

R (4.3.1) software was employed for all statistical analyses. Variables with missing values exceeding 20% were excluded from the study. For variables with missing values below 20%, multiple imputation was conducted using the MICE package. Patients meeting the inclusion criteria were randomly split into a training set (70%) and a validation set (30%).

## 3. Results

### 3.1 Baseline characteristics and clinical data

A total of 803 SAH patients meeting the inclusion and exclusion criteria were randomly divided into a training set (563 cases) and a validation set (240 cases) in a 7:3 ratio. Among them, 384 patients developed sepsis, while 419 did not. The demographic, clinical, and laboratory baseline characteristics of the patients in both groups are presented in Table 1.

**Table 1:** Baseline characteristics and clinical data.

Variables	trian data(N = 563)	validation data(N = 240)	*p*
Sepsis, n(%)			0.114
no	304 (54.0%)	115 (47.9%)	
yes	259 (46.0%)	125 (52.1%)	
age,Median(Q1,Q3)	61.00(50.00, 72.00)	60.00(53.00, 72.00)	0.333
gender, n(%)			0.533
female	315 (56.0%)	140 (58.3%)	
man	248 (44.0%)	100 (41.7%)	
BMI,Median(Q1,Q3)	26.72(23.20, 30.84)	27.58(23.94, 31.15)	0.216
race, n(%)			0.803
White	334 (59.3%)	140 (58.3%)	
Black	44 (7.8%)	15 (6.2%)	
Asian	21 (3.7%)	7 (2.9%)	
Hispanic/Latino	29 (5.2%)	15 (6.2%)	
Others	135 (24.0%)	63 (26.2%)	
hr,Median(Q1,Q3)	78.00(69.00, 89.00)	80.00(68.00, 90.25)	0.458
bps,Median(Q1,Q3)	129.00(114.00, 144.00)	128.00(113.00, 142.00)	0.474
bpd,Median(Q1,Q3)	69.00(61.00, 80.00)	69.00(59.00, 81.00)	0.735
bpm,Median(Q1,Q3)	84.00(75.00, 95.00)	85.00(75.00, 95.00)	0.951
rr,Median(Q1,Q3)	17.00(14.00, 20.00)	17.00(15.00, 20.00)	0.212
spo2,Median(Q1,Q3)	99.00(96.00, 100.00)	99.00(96.00, 100.00)	0.786
temperature,Median(Q1,Q3)	36.83(36.56, 37.11)	36.83(36.48, 37.17)	0.99
wbc,Median(Q1,Q3)	11.50(8.90, 14.40)	11.50(8.60, 15.53)	0.593
rbc,Median(Q1,Q3)	4.06(3.66, 4.49)	4.07(3.64, 4.44)	0.726
platelet,Median(Q1,Q3)	214.00(172.00, 263.50)	219.00(177.00, 264.00)	0.737
hemoglobin,Median(Q1,Q3)	12.30(11.20, 13.60)	12.20(11.00, 13.30)	0.376
rdw,Median(Q1,Q3)	13.40(12.90, 14.25)	13.45(12.78, 14.33)	0.862
sodium,Median(Q1,Q3)	139.00(137.00, 142.00)	140.00(137.00, 142.00)	0.591
potassium,Median(Q1,Q3)	3.90(3.60, 4.20)	3.90(3.60, 4.23)	0.106
chloride,Median(Q1,Q3)	105.00(102.00, 108.00)	105.00(102.00, 108.00)	0.257
glucose,Median(Q1,Q3)	132.00(111.00, 157.00)	129.50(112.00, 158.25)	0.737
aniongap,Median(Q1,Q3)	14.00(13.00, 17.00)	14.00(13.00, 17.00)	0.707
pt,Median(Q1,Q3)	12.30(11.50, 13.30)	12.40(11.40, 13.53)	0.586
ptt,Median(Q1,Q3)	27.80(25.25, 30.70)	27.50(25.40, 30.63)	0.876
Inr,Median(Q1,Q3)	1.10(1.00, 1.20)	1.10(1.00, 1.20)	0.752
ureanitrogen,Median(Q1,Q3)	13.00(10.00, 18.00)	14.00(10.00, 19.00)	0.25
creatinine,Median(Q1,Q3)	0.80(0.60, 1.00)	0.80(0.60, 0.92)	0.841
ventilation, n(%)			0.091
no	318 (56.5%)	120 (50.0%)	
yes	245 (43.5%)	120 (50.0%)	
embolism, n(%)			0.446
no	427 (75.8%)	188 (78.3%)	
yes	136 (24.2%)	52 (21.7%)	
Hypertension, n(%)			0.584
no	279 (49.6%)	124 (51.7%)	
yes	284 (50.4%)	116 (48.3%)	
Diabetes Mellitus, n(%)			0.979
no	493 (87.6%)	210 (87.5%)	
yes	70 (12.4%)	30 (12.5%)	
malignant tumor, n(%)			0.826
no	511 (90.8%)	219 (91.2%)	
yes	52 (9.2%)	21 (8.8%)	
Hyperlipidemia, n(%)			0.259
no	420 (74.6%)	188 (78.3%)	
yes	143 (25.4%)	52 (21.7%)	
CKD, n(%)			0.694
no	534 (94.8%)	226 (94.2%)	
yes	29 (5.2%)	14 (5.8%)	

BMI: Body Mass Index, hr: Heart Rate, bps: Systolic Blood Pressure, bpd: Diastolic Blood Pressure, bpm: mean arterial pressure, rr: Respiratory Rate, spo2: Oxygen Saturatio, wbc: White Blood Cell Count, rbc: Red Blood Cell Count, rdw: Red Cell Distribution Width, pt: Prothrombin Time, ptt: Partial Thromboplastin Time, inr: International Normalized Ratio, CKD: Chronic Kidney Disease

### 3.2 LASSO regression screening results

**Fig 1:**
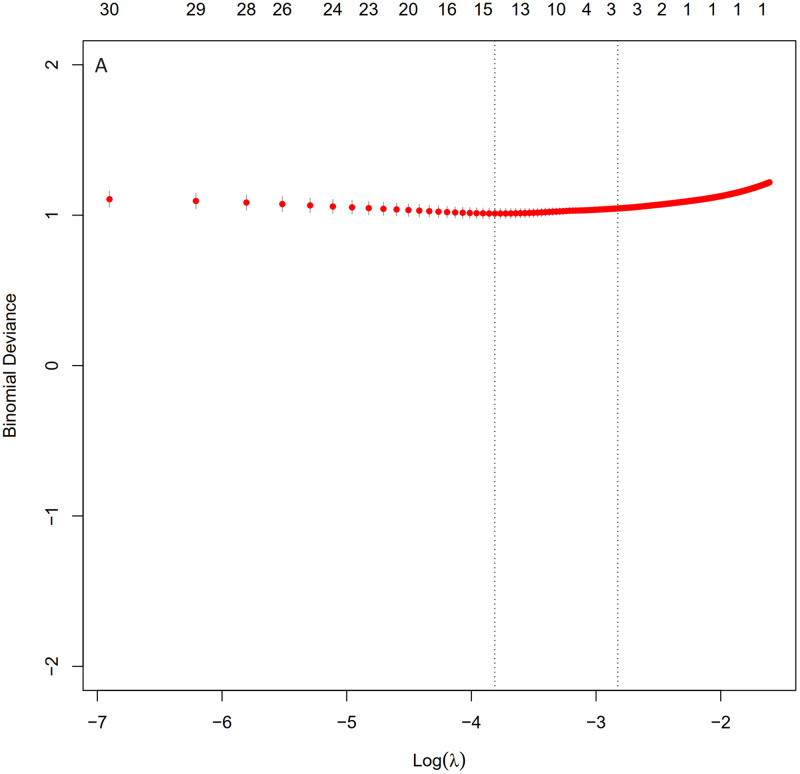
Selection of predictive variables using LASSO regression analysis and ten-fold cross-validation. The tuning parameter of LASSO regression (lambda) was selected based on the minimum criteria (left dashed line) and the one-standard error criteria (1-SE, right dashed line).

**Fig 2:**
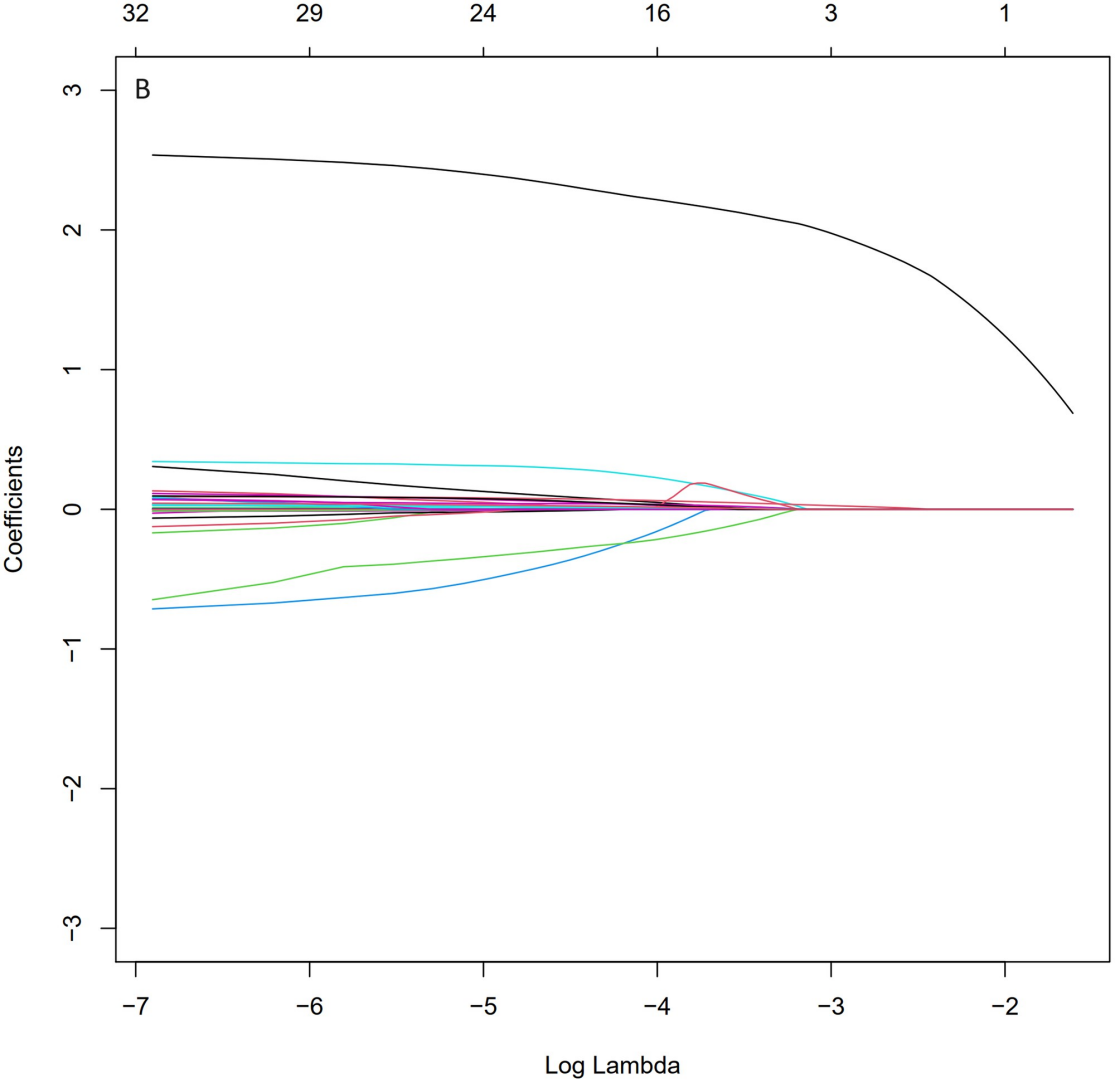
Selection of predictive variables using LASSO regression analysis and ten-fold cross-validation. Coefficient distribution was generated from the log(lambda) sequence. In this study, predictors were selected based on the 1-SE criteria (right dashed line), resulting in the selection of five non-zero coefficients. LASSO, least absolute shrinkage and selection operator; SE, standard error.

The LASSO (Least Absolute Shrinkage and Selection Operator) regression algorithm was utilized to identify the most significant predictive factors and prevent overfitting. The optimal parameter (Lambda) in the LASSO model was determined through ten-fold cross-validation with the minimum standard. The LASSO analysis revealed that mechanical ventilation, heart rate, respiratory rate, embolism, hyperlipidemia, malignant tumor, temperature, white blood cell count, and red blood cell count were the five identified risk factors for sepsis in SAH patients (Figs 1 and 2).

### 3.3 Multivariate logistic regression analysis of risk factors

Subsequently, a multivariate logistic regression analysis was conducted using the risk factors identified by LASSO as independent variables and the occurrence of sepsis as the dependent variable. After excluding variables with P>0.05, five factors were found to be significantly associated with sepsis occurrence: mechanical ventilation at ICU admission, hyperlipidemia at ICU admission, temperature at ICU admission, elevated white blood cell count at ICU admission, and decreased red blood cell count at ICU admission (Table 2).

**Table 2:** Multivariable logistic regression identifies optimal predictive factors for diagnosing sepsis in SAH patients.

Variables	Univariate analysis		Multivariate analysis	
	OR(95%CI)	p	OR(95%CI)	p
Temperature	1.346(1.031, 1.758)	0.029	1.443(1.032, 2.017)	0.032
Wbc	1.131(1.087, 1.177)	<0.001	1.126(1.070, 1.185)	<0.001
Rbc	0.724(0.555, 0.946)	0.018	0.618(0.433, 0.884)	0.008
Ventilation				
No	Reference		Reference	
Yes	13.571(9.039,20.375)	<0.001	12.130(7.863,18.711)	<0.001
Hyperlipidemia				
No	Reference		Reference	
Yes	1.526(1.042, 2.234)	0.03	2.202(1.346, 3.602)	0.002

The optimal predictive factors identified were the use of mechanical ventilation, hyperlipidemia, temperature, white blood cell count, and red blood cell count.wbc: White Blood Cell Count, rbc: Red Blood Cell Count

### 3.4 Establishment of the nomogram and evaluation of the diagnostic model

**Fig 3:**
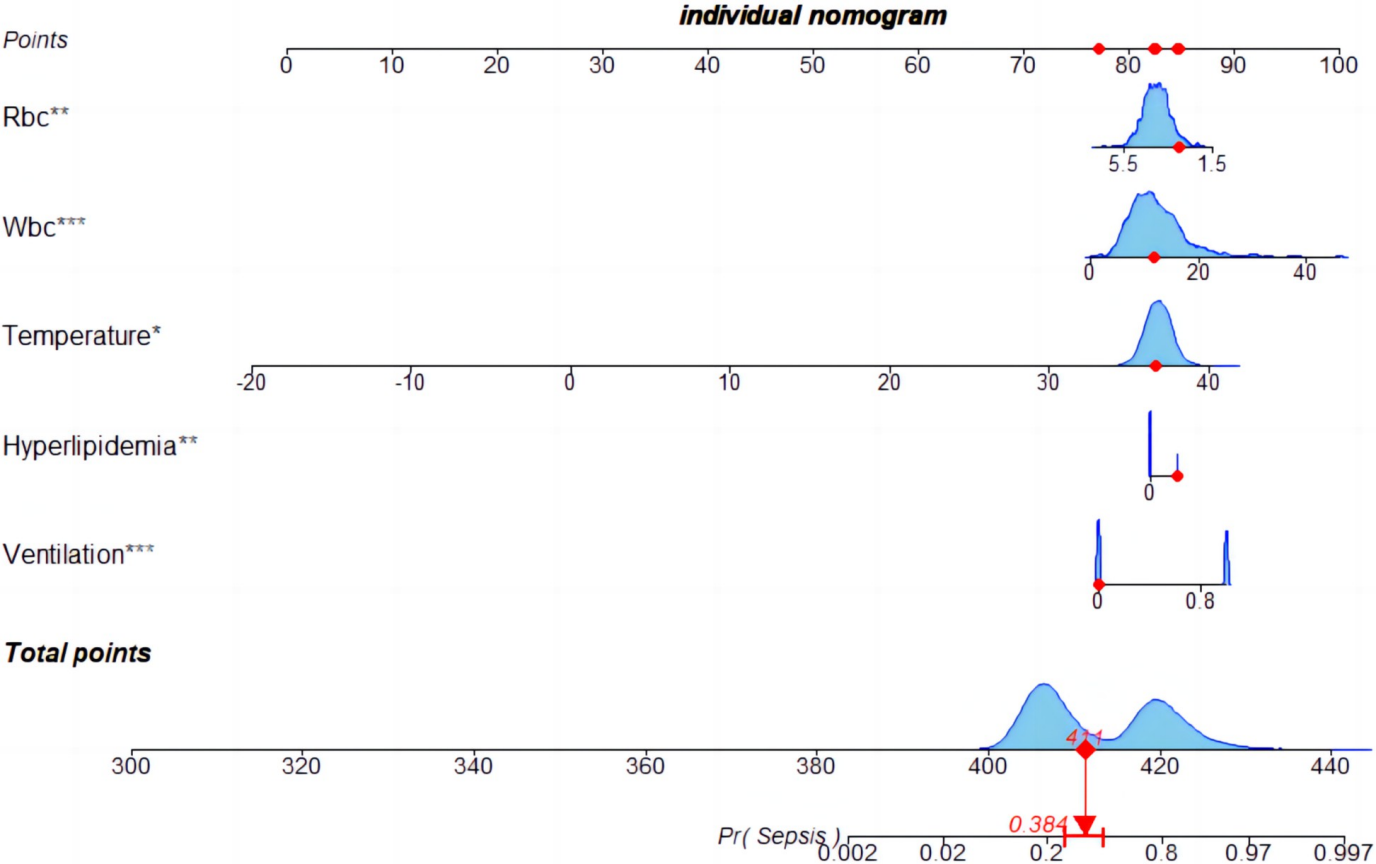
Nomogram for predicting the probability of sepsis in SAH patients in the training cohort. To obtain the corresponding score for each variable, draw a vertical line upward from the points axis. The total score at the bottom of the nomogram represents the probability of sepsis based on the sum of all variable scores.

**Fig 4:**
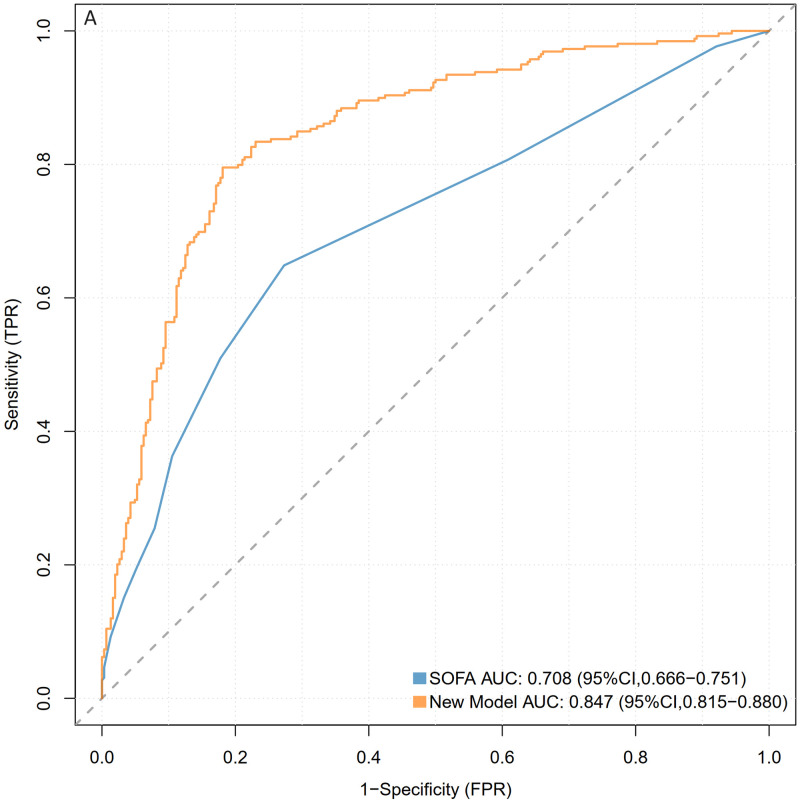
ROC curves of the nomogram model and the SOFA model. Fig 4 represents the training set.

**Fig 5:**
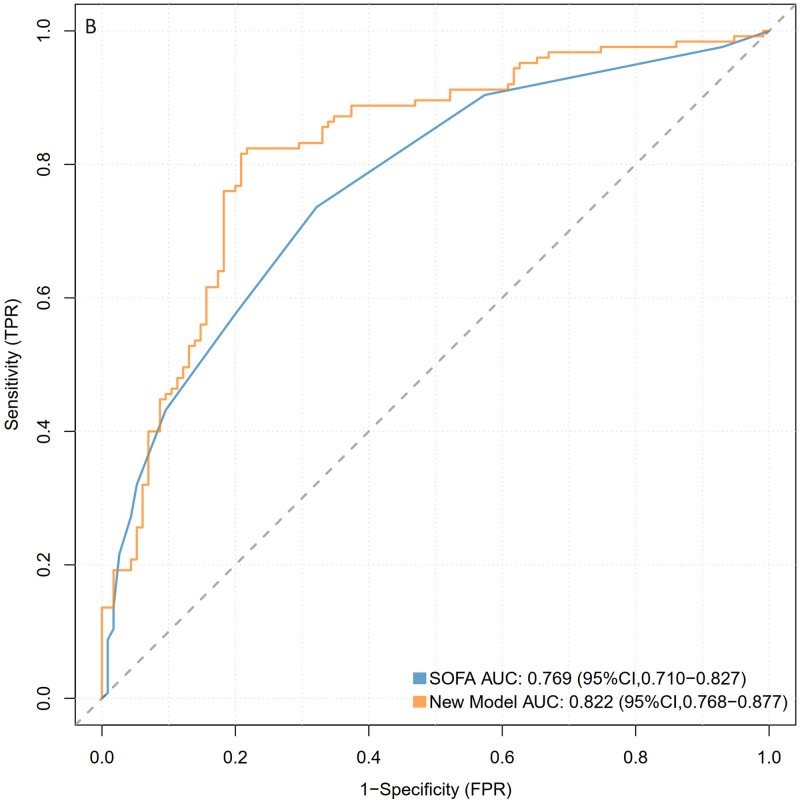
ROC curves of the nomogram model and the SOFA model. Fig 5 represents the validation set.

A nomogram was constructed using the five variables identified from the LASSO regression analysis (mechanical ventilation, hyperlipidemia, temperature, white blood cell count, and red blood cell count) as predictive factors, with the occurrence of sepsis as the clinical outcome (Fig 3). The diagnostic and predictive capability of the nomogram was compared with the SOFA score for sepsis. The AUC values of the nomogram in the training and validation sets were 0.854 (95% CI = 0.822–0.886) and 0.824 (95% CI = 0.770–0.877), respectively, both higher than those of the SOFA scoring system (Figs 4 and 5). Compared to the SOFA score, the NRI values in the training set were 0.2433 (95% CI = 0.1534–0.3331) and the IDI values were 0.2572 (95% CI = 0.2144–0.2999); in the validation set, the corresponding NRI value was 0.1757 (95% CI = 0.047–0.3043) and the IDI value was 0.1994 (95% CI = 0.1326–0.2662)(Table 3). These results indicate that our model has a superior ability to identify sepsis in SAH patients.

**Table 3:** Performance comparison of two models predicting sepsis occurrence in SAH patients.

Predict Model		AUROC	P-value	NRI(categorical)	P-value	NRI(continuous)	P-value	IDI	P-value
Training Set	Nomogram	0.847 [0.815–0.880]							
	SOFA	0.708 [0.666–0.751]	<0.001	0.2433 [0.1534–0.3331]	<0.001	0.95 [0.8047–1.0953]	<0.001	0.2572 [0.2144–0.2999]	<0.001
Validation Set	Nomogram	0.822 [0.768–0.877]							
	SOFA	0.769 [0.710–0.827]	<0.001	0.1757 [0.047–0.3043]	0.007	0.8932 [0.6693–1.1172]	<0.001	0.1994 [0.1326–0.2662]	<0.001

The P-value was calculated by comparing the results of nomogram with SOFA. SOFA, Sequential Organ Failure Assessment; AUROC, Area Under the ROC Curve; NRI, Net Reclassification Improvement; IDI, Integrated Discrimination Improvement.

**Fig 6:**
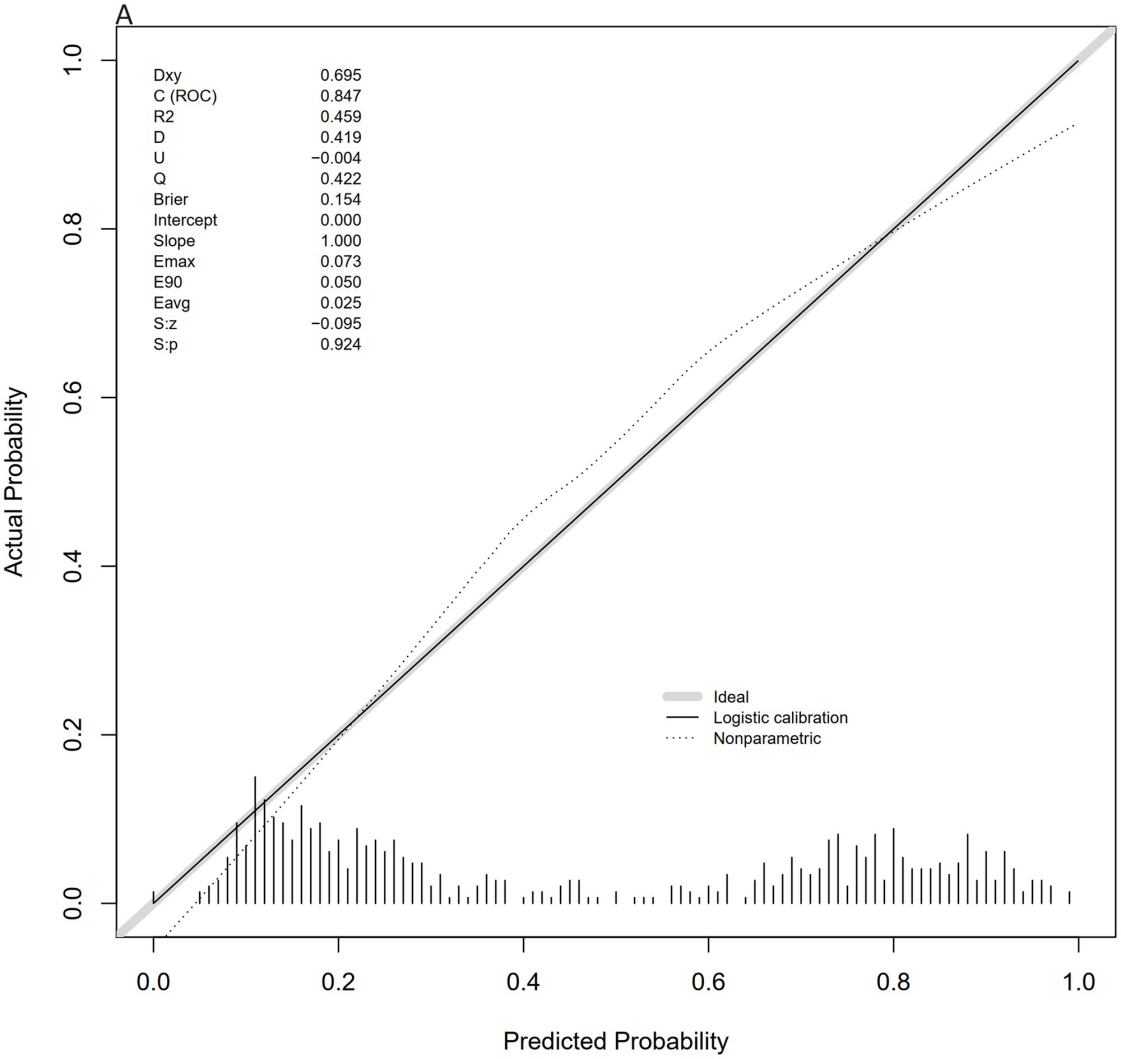
Calibration curves for the training and validation sets. Fig 6 shows the calibration curve for the training set.

**Fig 7:**
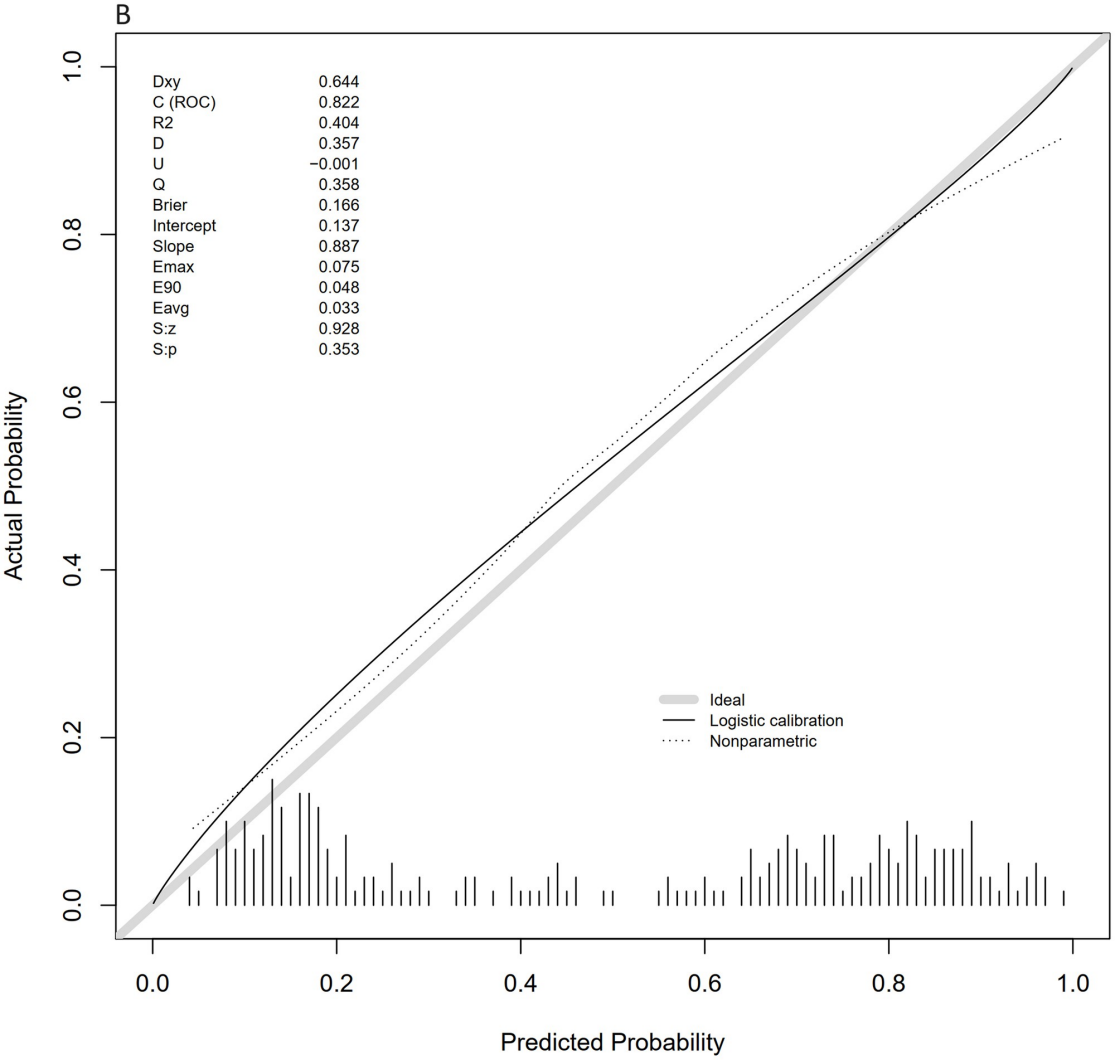
Calibration curves for the training and validation sets. Fig 7 shows the calibration curve for the validation set.

**Fig 8:**
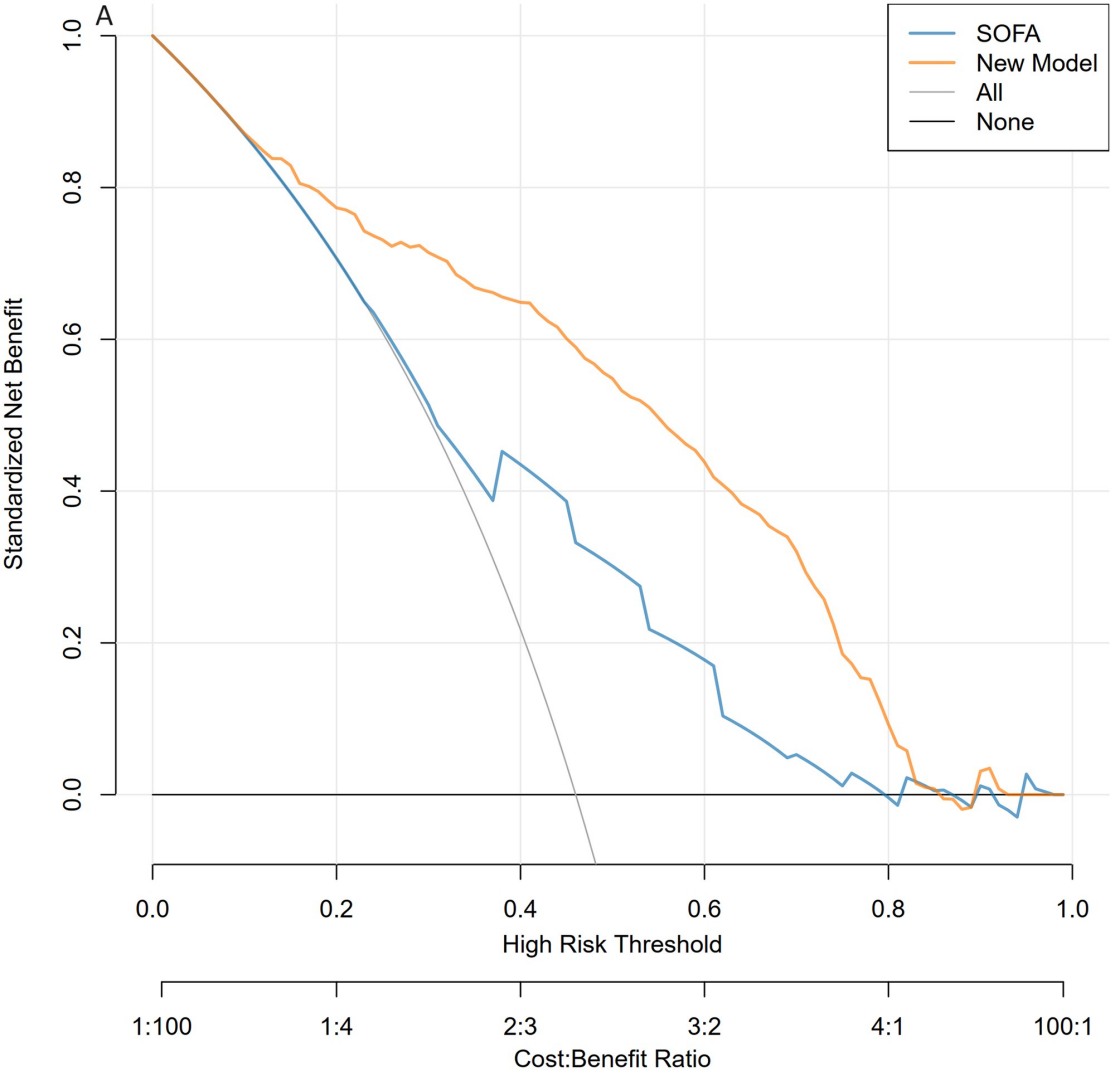
Decision curve analysis for the validation and training sets. The blue line represents the SOFA model, and the yellow line represents the nomogram model. Fig 8 shows Decision Curve analysis for the training sets.

**Fig 9:**
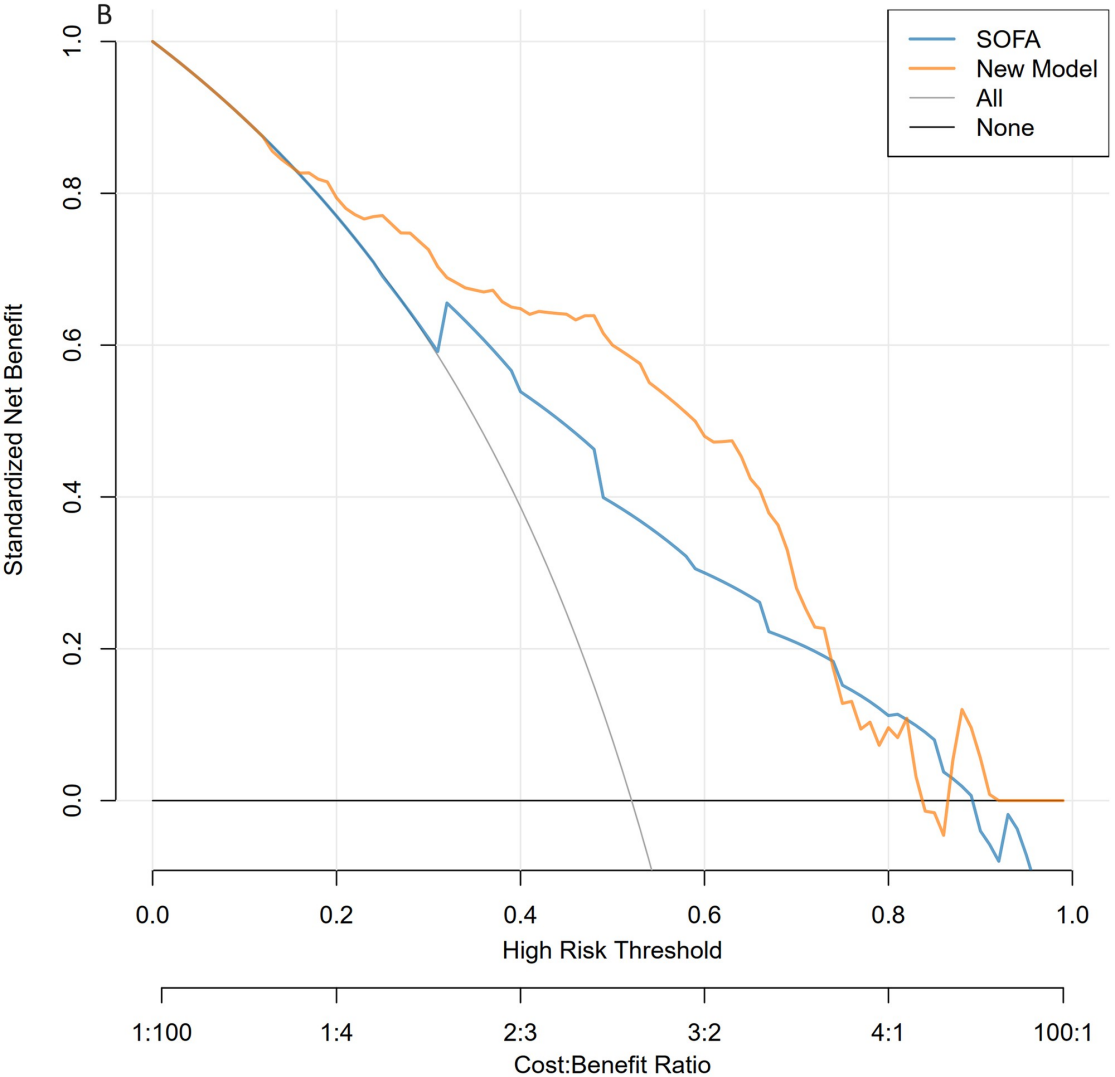
Decision curve analysis for the validation and training sets. The blue line represents the SOFA model, and the yellow line represents the nomogram model. Fig 9 shows Decision Curve analysis for the validation sets.

The calibration curves of the nomogram, shown in Figs 6 and 7, demonstrate that the calibration curves of the training and validation sets were almost diagonal. The C-index of the training set was 0.847 and the C-index of the validation set was 0.827, reflecting a high degree of predictive consistency in our model. The Hosmer-Lemeshow test results showed no statistical significance (training cohort: Χ^2^ = 6.124, P = 0.6333; validation cohort: Χ^2^ = 5.2974, P = 0.7254), indicating that the nomogram fit well with the data. Finally, the DCA (Decision Curve Analysis) curve illustrated the clinical applicability of the nomogram and compared it with the SOFA score (Figs 8 and 9). When the threshold probability of the two cohorts was between 0.08 to 0.85 and 0.13 to 0.83, the clinical diagnosis guided by our nomogram demonstrated higher net benefits compared to the currently used scoring system.

## 4. Discussion

Subarachnoid hemorrhage (SAH) is a critical neurological emergency frequently triggered by the rupture of cerebral aneurysms [14, 15]. Despite advancements in diagnosis and treatment, mortality and morbidity rates related to SAH remain high [16]. Sepsis, a common complication in SAH patients, exacerbates the condition and raises mortality risk [17]. Therefore, early identification and intervention for sepsis are crucial for enhancing the prognosis of SAH patients.

In this study, we examined data from 803 SAH patients in the MIMIC-IV database to create and validate a nomogram model for predicting sepsis risk. Our findings identified mechanical ventilation, hyperlipidemia, body temperature, white blood cell count, and red blood cell count as independent predictors of sepsis in SAH patients. Compared to the traditional SOFA scoring system [1], the nomogram model exhibited superior accuracy and reliability in predicting sepsis risk.

Mechanical ventilation is a common intervention in ICU patients, especially those with SAH [18], reflecting the severity of the patient’s condition and significant respiratory dysfunction. Studies indicate that mechanical ventilation is linked to a higher risk of sepsis, possibly due to the occurrence of ventilator-associated pneumonia (VAP) and other complications [19, 20]. VAP, a prevalent ICU infection, is usually caused by bacterial infections, triggering severe systemic inflammatory responses and sepsis [21, 22].

Hyperlipidemia is a recognized risk factor for various cardiovascular diseases [23]. Elevated levels of lipid biomarkers in sepsis indicate disruptions in lipid metabolism, potentially linked to inflammatory responses, immune regulation, and disease severity [24]. Increased triglycerides and fatty acids, which have inherent signaling properties, play a crucial role in this dysfunction, leading to tissue damage, organ failure, and immune cell dysfunction. These interconnected factors contribute to a complex interaction between prolonged inflammasome activation and disrupted lipid pro-resolving mediators. This imbalance is worsened by a state of low-grade endotoxemia and Damage-Associated Molecular Patterns (DAMPs), ultimately triggering an inappropriate inflammatory response [25]. Gram-negative bacterial sepsis induces hyperlipidemia by affecting liver sinusoidal endothelial cells (LSECs), resulting in impaired lipoprotein uptake and hypertriglyceridemia [26]. Despite this, studies suggest that hypolipidemia is linked to a poor prognosis in sepsis, which does not contradict our findings. Our results indicate that individuals with hyperlipidemia are more prone to developing sepsis in SAH [27].

Elevated body temperature is a significant factor associated with the development and prognosis of sepsis in patients with aneurysmal subarachnoid hemorrhage [28]. It serves as an indicator of infection, a marker for SIRS, and a diagnostic tool for early sepsis detection [29]. Clinicians should closely monitor body temperature in these patients to enable timely intervention and improve clinical outcomes [9]. Similarly, elevated WBC count is a significant factor linked to sepsis development and prognosis, serving as an indicator of infection, a marker for SIRS, and a diagnostic tool for early sepsis detection [30–32]. Red blood cells (RBCs) play a critical role in sepsis, with decreased RBC count seen in this condition due to factors like functional iron deficiency, decreased erythropoietin synthesis, infection, and inflammation [33, 34]. Pre-existing conditions such as cancer, liver disease, and renal impairment, along with new-onset multiple organ dysfunction, contribute to RBC loss [33]. Pathogen-induced hemolysis and immune reactions can also affect RBC morphology and rheology, leading to increased clearance by the spleen or liver. Additionally, volume resuscitation-induced hemodilution and blood loss from repeated phlebotomy further reduce RBC count [35].

The nomogram model outperforms the traditional SOFA scoring system in evaluating sepsis risk in SAH patients for several reasons. A significant advantage is that SAH patients often need sedation during mechanical ventilation, which can affect the Glasgow Coma Scale (GCS) score used in the SOFA score. In contrast, the nomogram model does not depend on GCS assessment, removing this complicating factor. This exclusion improves the nomogram’s predictive accuracy, making it a more dependable tool for identifying high-risk patients and guiding personalized treatment strategies. By incorporating multiple independent predictors, the nomogram offers clinicians a simple and intuitive method for assessing sepsis risk in SAH patients, ultimately enhancing the effectiveness of clinical decision-making.

However, This study has several limitations that warrant discussion. Firstly, the retrospective nature of our study introduces potential biases. Selection bias may have occurred due to the inclusion criteria of the MIMIC-IV database, which may not fully represent the general SAH population. Information bias is another concern, as the accuracy and completeness of the data depend on the quality of medical record documentation. Secondly, the MIMIC-IV database, while extensive, has its own limitations. It represents data from a single, tertiary care center in the United States, which may limit the generalizability of our findings to other healthcare settings or geographical regions. The database may also lack certain clinical variables that could be relevant to SAH prognosis and sepsis development. Thirdly, our study’s timeframe (2008–2019) spans a period during which clinical practices and sepsis definitions evolved. The Sepsis-3 criteria, published in 2016, may not have been uniformly applied throughout our study period, potentially affecting the consistency of sepsis diagnosis. Fourthly, while our nomogram showed improved predictive performance compared to the SOFA score, the magnitude of this improvement was modest. This raises questions about the clinical significance and cost-effectiveness of implementing a new predictive tool in practice. Lastly, the MIMIC-IV database offers a wealth of real-world data, further validation through prospective, multicenter studies is necessary to confirm the clinical utility of the nomogram model.

Based on the study’s findings, clinicians should intensify monitoring of SAH patients, especially those on mechanical ventilation, with hyperlipidemia, elevated body temperature, increased white blood cell count, and decreased red blood cell count, while remaining vigilant for sepsis. Early detection and management of sepsis can significantly enhance patient outcomes. Additionally, fostering multidisciplinary collaboration among neurology, critical care, infectious disease, and nursing teams is essential for improving the prognosis of SAH patients.

## 5. Conclusion

This study introduces a novel tool for early sepsis prediction in patients with subarachnoid hemorrhage (SAH) and provides valuable insights for clinical decision-making. Further research is needed to validate and enhance the use of this nomogram in improving the outcomes of SAH patients.

## Supporting information

S1 Data (CSV)
